# Identification of genes essential for pellicle formation in *Acinetobacter baumannii*

**DOI:** 10.1186/s12866-015-0440-6

**Published:** 2015-06-06

**Authors:** Sarah K. Giles, Uwe H. Stroeher, Bart A. Eijkelkamp, Melissa H. Brown

**Affiliations:** School of Biological Sciences, Flinders University, GPO Box 2100, 5001 Adelaide, SA Australia; Research Centre for Infectious Diseases, School of Biological Sciences University of Adelaide, Adelaide, Australia

**Keywords:** Cyclic AMP, Motility, Virulence, Biofilm, Hydrophobicity

## Abstract

**Background:**

*Acinetobacter baumannii* is an opportunistic pathogen, which has the ability to persist in the clinical environment, causing acute and chronic infections. A possible mechanism contributing to survival of *A. baumannii* is its ability to form a biofilm-like structure at the air/liquid interface, known as a pellicle. This study aimed to identify and characterise the molecular mechanisms required for pellicle formation in *A. baumannii* and to assess a broad range of clinical *A. baumannii* strains for their ability to form these multicellular structures.

**Results:**

Random transposon mutagenesis was undertaken on a previously identified hyper-motile variant of *A. baumannii* ATCC 17978 designated 17978hm. In total three genes critical for pellicle formation were identified; *cpdA*, a phosphodiesterase required for degradation of cyclic adenosine monophosphate (cAMP), and A1S_0112 and A1S_0115 which are involved in the production of a secondary metabolite. While motility of the A1S_0112::Tn and A1S_0115::Tn mutant strains was abolished, the *cpdA*::Tn mutant strain displayed a minor alteration in its motility pattern. Determination of cAMP levels in the *cpdA*::Tn strain revealed a ~24-fold increase in cellular cAMP, confirming the role CpdA plays in catabolising this secondary messenger molecule. Interestingly, transcriptional analysis of the *cpdA*::Tn strain showed significant down-regulation of the operon harboring the A1S_0112 and A1S_0115 genes, revealing a link between these three genes and pellicle formation. Examination of our collection of 54 clinical *A. baumannii* strains revealed that eight formed a measurable pellicle; all of these strains were motile.

**Conclusions:**

This study shows that pellicle formation is a rare trait in *A. baumannii* and that a limited number of genes are essential for the expression of this phenotype. Additionally, an association between pellicle formation and motility was identified. The level of the signalling molecule cAMP was found to be controlled, in part, by the *cpdA* gene product, in addition to playing a critical role in pellicle formation, cellular hydrophobicity and motility. Furthermore, cAMP was identified as a novel regulator of the operon A1S_0112-0118.

**Electronic supplementary material:**

The online version of this article (doi:10.1186/s12866-015-0440-6) contains supplementary material, which is available to authorized users.

## Background

*Acinetobacter baumannii* is an opportunistic Gram-negative human pathogen that causes severe nosocomial infections primarily in immune-compromised patients; disease states include pneumonia, meningitis and bacteraemia [1–5]. The bacterium can be found in intensive care units and is frequently isolated from medical devices [6–9]. The capacity to survive in these environments is possibly due to the ability of this organism to survive desiccation and resist a broad spectrum of antibiotics and disinfectants. As a result, *A. baumannii* has entrenched itself in the clinical environment [10–12].

Given the occurrence of *A. baumannii* in intensive care units and its high level of intrinsic and adapted resistance, there is increased pressure to identify novel drug targets. Therefore, understanding the molecular mechanisms contributing to virulence in *A. baumannii* is essential. Similar to numerous other organisms, *A. baumannii* has the ability to grow a multicellular structure known as a biofilm (reviewed by Longo *et al*. 2014) [13] of which the pellicle is a specialised form. The pellicle is localised to the interface between air and liquid, and is a structure of connected cells surrounded by a matrix of extracellular polymeric substance [14, 15]. The formation of a pellicle allows aerobic bacteria to have access in a stagnant environment to high levels of oxygen and nutrients [16]. The pellicle can increase bacterial survival under antibiotic pressure as well as contribute to longer than usual persistence in a hostile environment, such as under drying conditions [17–19].

There are numerous molecular mechanisms potentially involved in pellicle formation by *A. baumannii*. One such mechanism is the *csuA/BABCDE* pilus chaperone-usher assembly system which is responsible for the production of pili and is essential in the formation of a biofilm on solid surfaces [2, 9, 20]. In a study on *Bacillus subtilis*, 288 regulatory and potential regulatory genes were associated with pellicle formation [21]. Recent analyses of *A. baumannii* bacteria in the pellicle state have revealed alterations in the expression of at least 52 membrane proteins (32 up-regulated and 20 down-regulated) [22, 23]. These 52 proteins were found to be involved in; iron uptake systems, lipid and carbohydrate transport, cellular metabolism, starvation, in addition to porins and pili.

As bacteria encounter a wide range of environmental conditions, there is a requirement to respond to these changes, such as changing from a planktonic state to living within a biofilm and/or pellicle; this can involve deployment of a number of signalling molecules [24, 25]. Cyclic adenosine monophosphate (cAMP) is a ubiquitous signalling molecule used in conjunction with a variety of regulators for modulating gene expression in both prokaryotes and eukaryotes [26, 27]. In bacteria, it has been found to alter multiple virulence characteristics. For example, in *Vibrio cholerae* cAMP has been implicated in the regulation of cholera toxin, the toxin co-regulated pilus and other pathogenic factors [28]. In *Serratia marcesens* the importance of the cAMP receptor protein (CRP complex) in the formation of biofilm has been established [26]. In this case the type I fimbriae-dependant biofilm is tightly regulated and a deletion in the phosphodiesterase gene was found to increase cAMP within the bacteria cell and consequently decrease biofilm formation. Conversely, when increased copies of the phosphodiesterase gene are introduced into the bacterial cell, the bacteria enter into what is described as a “hyper biofilm” state [26]. Similarly in *Pseudomonas aeruginosa*, cAMP has been identified as a key component in biofilm formation, where increased levels of cAMP can inhibit adherence capabilities [29]. Furthermore, in this organism cAMP functions as a co-factor of the virulence factor regulator Vfr, where the cAMP-Vfr complex regulates type III secretion systems involved in exporting toxins and other compounds [30–32]. This complex also regulates virulence factors such as type IV pili, the *las* quorum sensing system and exotoxin A [32, 33]. Although a *vfr*-like gene is present in the *A. baumannii* genome, its role in regulation of virulence factors and interaction with cAMP has not been reported.

The proteomic analyses by Marti *et al*. (2011) and Nait Chabane *et al*. (2014) suggested that pellicle formation in *A. baumannii* is not the result of a single gene or operon [22, 23]. Instead, it is likely that pellicle formation is multifactorial, requiring surface-exposed molecular structures and an adequate level of transcriptional regulation. The aim of this study is to provide insight into the genetic elements that contribute to the ability of *A. baumannii* to migrate to a surface and form a pellicle as well as to assess the prevalence of pellicle formation in a number of clinically-relevant *A. baumannii* strains.

## Results and discussion

### Pellicle formation is a rare trait in clinical *A. baumannii* strains

In this study, we have defined the pellicle as a robust layer of connected cells covering the surface of a liquid. To assess the prevalence of pellicle formation, our collection of clinical *A. baumannii* strains [34] was screened for this phenotype. Pellicle formation was assessed using glass and polypropylene tubes, as these materials are used for a number of implements within the hospital environment and have different surface properties, which may influence the initial attachment and formation of a pellicle. Preliminary assessment of pellicle biomass was conducted at growth temperatures of 25 °C and 37 °C (data not shown), which revealed that pellicle growth predominantly occurred at 25 °C; these results coincide with Marti *et al*. (2011) [35], as such, all subsequent analyses were performed at 25 °C. Of the 54 clinical strains tested only eight formed a robust pellicle, suggesting that this is not a ubiquitous phenotype (Table 1). These eight strains produced varying amounts of total pellicle biomass when comparing glass and polypropylene strata (Table 1). Pellicle formation was categorised into three groups dependent on the amount of pellicle material measured at OD_600_ where a; “small pellicle” is considered >0.19, “moderate pellicle” is considered >0.20 and <0.50 and “well-developed pellicle” is >0.51. In glass tubes, strains ATCC 17978, 17978hm, WM98c, 04145027 and AYE produced a well-developed pellicle, while, AB0057, 11986752 and 2320495 produced a moderate to small pellicle. Analysis in polypropylene tubes showed that only two strains, 17978hm and 04145027, produced a strong pellicle, while four strains, ATCC 17978, 11986752, 2320495 and WM98c, produced a moderate to small pellicle. Both AYE and AB0057 formed a pellicle in glass tubes, but failed to form a measurable pellicle in polypropylene tubes. Hence, the ability to form a pellicle was not conserved across glass and polypropylene surfaces. These observed differences could be due to a number of factors related to the specific adherence strategies of each strain, such as the production of pili and other surface-exposed macromolecules. A number of strains, 6772166, 6856775, PW01c, 6877889 and 6870155 produced a weak surface film that could not be extracted using our protocol, attempts to do so by other means such as draining the planktonic growth resulted in destruction of this weak surface film. Structures such as pilin-like proteins and exopolysaccharides have been shown to be present in pellicles [23] and it is possible that these structures are expressed at a reduced level in these strains resulting in only a weak surface film. Interestingly, all pellicle forming strains and those that formed a weak surface film have been shown to be motile [34]. This finding highlights the possibility that the molecular mechanisms involved in motility and pellicle formation are linked.

**Table 1:** Pellicle formation in glass and polypropylene, cell hydrophobicity, motility status and isolation site

*A. baumannii* isolate	Glass (OD_600_)	Polypropylene (OD_600_)	Hydrophobicity (%)	Motility	Isolation site
Pellicle forming *A. baumannii* strains					
ATCC 17978	0.65 ± 0.16	0.44 ± 0.08	32 ± 0.25	+	Meningitis
17978hm	1.03 ± 0.01	0.94 ± 0.02	42 ± 0.19	+	Meningitis
11986752	0.13 ± 0.01	0.17 ± 0.04	98 ± 0.01	+	Pus
2320495	0.35 ± 0.08	0.28 ± 0.06	98 ± 0.03	+	Pus
WM98c	1.01 ± 0.02	0.37 ± 0.08	94 ± 0.10	+	Unknown
04145027	0.62 ± 0.00	0.79 ± 0.04	100 ± 0.00	+	Vaginal
AYE	0.65 ± 0.11	Not detected	92 ± 0.07	+	Urinary
AB0057	0.47 ± 0.06	Not detected	0	+	Blood
Surface film forming *A. baumannii* strains					
6772166	BD^*a*^	BD	0	+	Pus
685775	BD	BD	0	+	Sputum
PW01c	BD	BD	0	+	Unknown
6877889	BD	BD	9 ± 0.09	+	Sputum
6870155	BD	BD	5 ± 0.07	+	Sputum

^***a***^ BD = below detection level

### Hydrophobicity across the clinical *A. baumannii* strains is not directly linked to pellicle formation ability

**Fig. 1:**
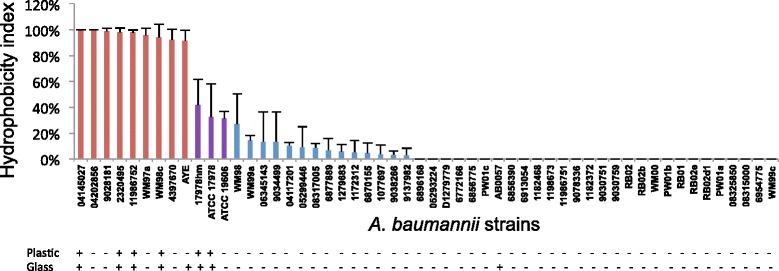
Hydrophobicity of *A. baumannii* strains. Cell surface hydrophobicity was assessed by the affiliation of the bacterial cell to xylene. Strains with a hydrophobic index above 70 % are shown in red, those above 30 % are in purple and those below 30 % are in blue. Those strains where no bar is visible are completely hydrophilic. The ability of each strain to form a pellicle in glass or polypropylene is shown as (+ or -) below the hydrophobic index. Bars indicate the standard deviation of three separate experiments

In a number of bacterial species, including *A. baumannii*, a positive association between cell surface hydrophobicity, biofilm formation and adherence capabilities has been reported [36–38]. We evaluated our set of clinical *A. baumannii* strains for cell surface hydrophobicity and identified major differences (Fig. 1). Using the divisions of the hydrophobicity index as established by Pour *et al*. (2011) [36], 42 of the strains displayed a hydrophilic character, nine were found to have a strong hydrophobic character and three showed an intermediate level of cell surface hydrophobicity. Of the eight strains that formed a pellicle in glass (Table 1), five (11986752, 2320495, WM98c, 04145027 and AYE) were found to have a strong hydrophobicity index of >70 %, two (ATCC 17978 and 17978hm) showed an intermediate hydrophobic character and AB0057 was found to be hydrophilic. There are numerous proteins embedded within the outer membrane of Gram-negative bacteria that have a variety of functions including pili for adherence and possibly pellicle formation as well as other physical characteristics. Surface proteins can alter the surface chemistry of a cell and affect the surface hydrophobicity [39, 40]. The aforementioned results suggest a high level of correlation between the ability to form an extractable pellicle and an increase in cell surface hydrophobicity. The level of hydrophobicity may have an influence on what surface and how strongly bacteria interact due to a variety of proteins on the cell surface [41, 42]. Interestingly, the strains identified as producing a weak surface film all show a hydrophilic nature.

### A1S_0112, A1S_0115 and *cpdA* are essential for pellicle formation in *A. baumannii* 17978hm

To further investigate the pellicle phenotype, we used our previously characterised *A. baumannii* 17978hm strain (harboring an insertion in *hns*) [43], which has the ability to produce increased amounts of pellicle material compared to the ATCC 17978 parent strain. This strain was chosen as it allows good discrimination between the pellicle and non-pellicle phenotype allowing ready identification in large scale screening procedures. To identify genes associated with this potential virulence factor a transposon bank of random mutants was constructed in the 17978hm strain using the plasmid pLOF mini::Tn*10*:*gfp*:*kan* [44]*.* Approximately 4000 individual transposon insertion mutants were examined in microtitre trays for their ability to form a pellicle. Initial screening identified nine transposon insertion mutants that were incapable of pellicle formation. Subsequent evaluation of these strains in polypropylene and glass tubes confirmed the complete loss of the pellicle phenotype (data not shown). The exact insertion site of the individual transposon insertions were identified by cloning the kanamycin resistance gene associated with the transposon (mini::Tn*10*:*gfp*:*kan*) and subsequent sequencing. Insertions were found to be within three genes: A1S_0249 (*cpdA*), a cAMP phosphodiesterase; A1S_0112, acyl-CoA synthetase/adenosine monophosphate (AMP) acid ligase II; and A1S_0115, which is annotated to be involved in amino acid adenylation. The precise insertion site of the transposon is given as the nucleotide position relative to the *A. baumannii* ATCC 17978 genome sequence (GenBank CP000521) (Additional file 1). Seven transposon insertions were identified to be within the A1S_0115 gene. A single representative of each of the three distinct insertions, designated transposon mutants 1, 2 and 5, was taken for further investigation (Additional file 1).

**Fig. 2:**
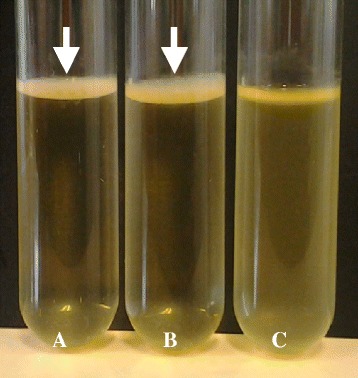
Pellicle formation by *A. baumannii* 17978hm parent, *cpdA*::Tn(0249) and *cpdA*::Tn(1266) strains. *A. baumannii* strains were grown in Luria-Bertani broth and analysed after a 72 h incubation at 25 °C in the absence of light. Pellicle material was assessed visually. (**a**) 17978hm parent strain; (**b**) *cpdA*::Tn(0249) complemented mutant strain showing developed pellicles at the interface between the liquid and air; and (**c**) *cpdA*::Tn(1266) mutant strain negative for pellicle formation. Arrows identify the pellicle material

Transposon mutant 1 was identified as containing an insertion within *cpdA*, *cpdA*::Tn. Bacteria finely modulate intracellular cAMP levels through adenylate cyclases (synthesis) and cAMP phosphodiesterases, such as CpdA, which converts cAMP to AMP (degradation) [45]. Therefore, as the insertional inactivation of *cpdA* led to a loss of pellicle, this insertion identified the requirement for cAMP regulation in this process. To confirm that the observed phenotype can be attributed to disruption of *cpdA*, the *cpdA*::Tn mutant strain was complemented by cloning a wild-type copy of *cpdA* into the empty shuttle vector pWH1266 [46], producing pWH0249. The pWH0249 plasmid was subsequently electroporated into the *cpdA*::Tn mutant strain generating the strain *cpdA*::Tn(0249) (Table 2). This strain was found to successfully produce a pellicle in contrast to both the *cpdA*::Tn mutant strain or the *cpdA*::Tn control strain harboring the empty pWH1266 vector, *cpdA*::Tn(1266) (Table 2; Fig. 2).

**Table 2:** A. baumannii 17978hm mutant derivatives constructed in this study

Abbreviated name	Description^*a*^
*cpdA*::Tn	Tn*10*:*gfp*:*kan* insertion in *cpdA,* kan^R^
*cpdA*::Tn(0249)	Tn*10*:*gfp*:*kan* insertion in *cpdA* containing pWH0249, kan^R^, amp^R^
*cpdA*::Tn(1266)	Tn*10*:*gfp*:*kan* insertion in *cpdA* containing shuttle vector pWH1266, kan^R^, amp^R^, tet^R^
A1S_0112::Tn	Tn*10*:*gfp*:*kan* insertion in A1S_0112, kan^R^
A1S_0112::Tn(0112)	Tn*10*:*gfp*:*kan* insertion in A1S_0112 containing pWH0112, kan^R^, amp^R^
A1S_0112::Tn(1266)	Tn*10*:*gfp*:*kan* insertion in A1S_0112 containing shuttle vector pWH1266, kan^R^, amp^R^, tet^R^
A1S_0115::Tn	Tn*10*:*gfp*:*kan* insertion in A1S_0115, kan^R^
A1S_0115::Tn(0115)	Tn*10*:*gfp*:*kan* insertion in A1S_0115 containing pWH0115, kan^R^, amp^R^
A1S_0115::Tn(1266)	Tn*10*:*gfp*:*kan* insertion in A1S_0115 containing shuttle vector pWH1266, kan^R^, amp^R^, tet^R^

^*a*^ kan^R^, kanamycin resistance at 50 μg/mL; amp^R^, ampicillin resistance at 100 μg/mL; tet^R^, tetracycline resistance at 12.5 μg/mL

**Fig. 3:**
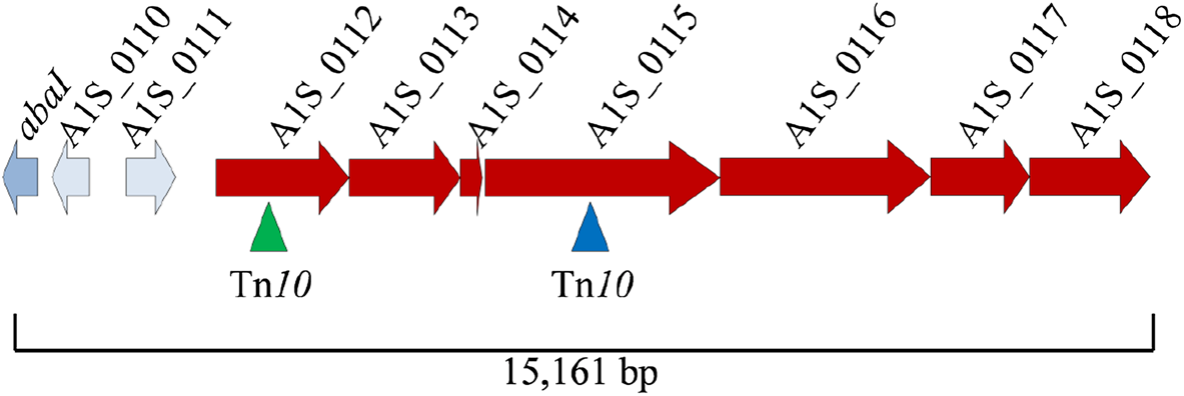
Operon with transposon insertion sites within A1S_0112 and A1S_0115 identified. The genetic organisation of the A1S_0112-0118 operon is shown with the arrows representing open reading frames and depict the direction of transcription. The location of the transposon (Tn*10*) insertions in A1S_0112 (green triangle) and A1S_0115 (blue triangle) are shown. Flanking genes illustrate the position of the operon within the chromosome adjacent to the quorum sensing gene *abaI,* A1S_0110 hypothetical protein and A1S_0111 transcriptional regulator; the total size of the region shown is 15,161 bp in length

Transposon mutant 2 A1S_0112::Tn and transposon mutant 5 A1S_0115::Tn have insertions within the open reading frame (ORFs) A1S_0112 and A1S_0115, which are located in a large operon comprised of seven genes, A1S_0112-0118 (Fig. 3). This operon has previously been associated with cell motility, potentially by production and secretion of a biosurfactant via a type-I secretion system or another mechanism [47, 48]. A recent report has shown that the genes within this operon are significantly up-regulated in biofilm cells in *A. baumannii* ATCC 17978 and a site-specific knockout of A1S_0114 abolished biofilm formation [49]. Efforts to complement the A1S_0112::Tn and A1S_0115::Tn transposon mutant strains with copies of the wild-type genes cloned into pWH1266 (Table 2), were unsuccessful possibly due to polar effects of the transposon insertions. In order to ensure that the lack of complementation was not due to a lack of transcription of our complementing genes, we undertook qRT-PCR that revealed adequate transcription occurred for the wild-type A1S_0112 and A1S_0115 genes cloned into the empty pWH1266 vector (Table 3, Additional file 2). As mentioned, pellicle formation was not restored in these mutants, possibly because, these ORFs are part of an operon which must be transcribed as a polycistronic message making complementation of these mutants not possible. This is supported by the work of Clemmer *et al.* (2011) who previously isolated transposon mutants in this region which abolished motility and due to the nature of this operon were also unable to complement the mutants [47].

**Table 3:** Fold change in gene expression of transposon mutant strains compared to the 17978hm parent strain

Strain	A1S_0112*	A1S_0115*	A1S_0118*
*cpdA*::Tn	-2.8 ± 0.21	ND	ND
*cpdA*::Tn(0249)	1.5 ± 0.44	ND	ND
*cpdA*::Tn(1266)	-5.6 ± 0.032	ND	ND
A1S_0112::Tn	NA	-335.46 ± 1.42	-28.44 ± 1.33
A1S_0112::Tn(0112)	-0.49 ± 1.39	-669.37 ± 1.24	-51.63 ± 1.70
A1S_0112::Tn(1266)	-171.25 ± 1.68	-325.53 ± 1.22	-85.43 ± 1.46
A1S_0115::Tn	-1.14 ± 1.59	NA	-83.29 ± 1.20
A1S_0115::Tn(0115)	-4.1 ± 1.09	-1.16 ± 1.63	-28.97 ± 1.54
A1S_0115::Tn(1266)	-0.99 ± 1.32	-347.29 ± 1.52	-72.50 ± 1.14

NA = not applicable

ND = not done

* = data from three biological replicates

### Characterisation of pellicle-deficient mutant strains *cpdA*::Tn, A1S_0112::Tn and A1S_0115::Tn

**Fig. 4:**
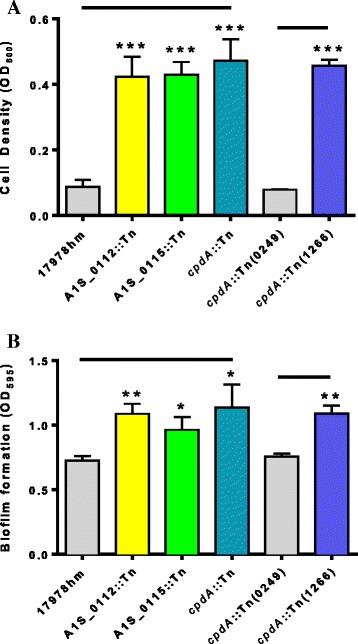
Planktonic growth and biofilm formation in polypropylene tubes of the transposon mutants and 17978hm parent strain. Cells were cultured in Luria Bertani media without shaking at 37 °C in polypropylene tubes for 72 h. (**a**) Planktonic growth was assessed by removal of 1 mL of media and the optical density measured at OD_600_. All three transposon insertion mutant strains showed a dramatic increase of planktonic growth; 4-fold greater than the 17978hm parent strain. Complementation of the *cdpA* mutation in the *cpdA*::Tn(0249) strain returned the phenotype to wild-type levels. (**b**) The level of biofilm produced was assessed by crystal violet staining. Transposon insertion mutant strains had an increased biomass of the surface/liquid biofilm by approximalty 30 % compared to that of the 17978hm parent. The *cpdA*::Tn(0249) mutant strain that contains a wild-type copy of the gene, restored the biofilm phenotype to that of the parent. Bars indicate the standard deviation of three separate experiments. Asterisks indicate significant difference: one denotes a p-value <0.05, two a p-value <0.01 and three a p-value <0.001, as determined by a student *t*-test

The three transposon insertion mutant strains described above were examined for potential growth perturbations in Luria-Bertani (LB) liquid cultures. All three *A. baumannii* transposon mutant strains showed comparable growth to the parent 17978hm strain (Additional file 3), indicating that the loss of the pellicle is not due to a general growth defect. Since pellicle formation is only one type of biofilm, the ability to form a biofilm at the liquid-surface interface was also examined. The three transposon mutants were assessed for both their planktonic growth potential and their ability to form a biofilm on polypropylene (Fig. 4a and b). All transposon mutants showed an increase in planktonic growth compared to the parent and *cpdA*::Tn(0249) complemented derivative (p-value <0.001) (Fig. 4a). The transposon insertion mutants were able to form a significantly increased amount of biofilm in polypropylene tubes compared to the parent strain (*cpdA*::Tn, A1S_0115::Tn and A1S_0112::Tn; p-values of 0.05, 0.05 and 0.01, respectively) (Fig. 4b), while, the complemented *cpdA*::Tn(0249) mutant strain returned the phenotype to that of the parent (Fig. 4). Carriage of wild-type copies of A1S_0112 and A1S_0115 in their respective Tn mutant derivatives did not result in functional complementation (data not shown).

**Fig. 5:**
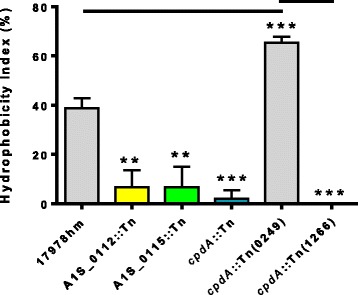
Hydrophobicity index for A1S_0112::Tn, A1S_0115::Tn and *cpdA*::Tn transposon insertion mutants. Hydrophobicity results were obtained by assessing the bacterial cell affiliation to xylene. The optical density of the cell suspension before (OD_inital_) and after (OD_final_) the addition of xylene was measured at 600 nm. All the transposon insertion mutant strains have reduced cell hydrophobicity compared to the parental strain. The *cpdA*::Tn(0249) mutant strain with a wild-type copy of *cpdA* has a hydrophobicity greater than the 17978hm parent strain. Bars indicate the standard deviation of three separate experiments. Asterisks indicate significant difference: two denotes a p-value <0.01 and three a p-value <0.001, as determined by a student *t*-test

The three pellicle deficient transposon mutants all displayed a >80 % reduction in cell surface hydrophobicity compared to the parent strain, to essentially a hydrophilic nature (*cpdA*::Tn, A1S_0112::Tn and A1S_0115::Tn; p-values of 0.001, 0.01 and 0.01, respectively) (Fig. 5). This indicates that hydrophobicity is affected when *cpdA*, A1S_0112 or A1S_0115 genes are insertionally inactivated. The presence of the wild-type *cpdA* gene in the *A. baumannii cpdA*::Tn(0249) derivative not only complemented the hydrophobicity character, but increased the hydrophobicity index to 60 % which is above the parent strain (p-value <0.001) (Fig. 5); this would indicate that cAMP is involved in modulating cell surface hydrophobicity. The increase in hydrophobicity above the level seen with the parent strain is likely due to a copy number effect of the wild-type *cpdA* determinant residing on the pWH1266 plasmid.

**Fig. 6:**
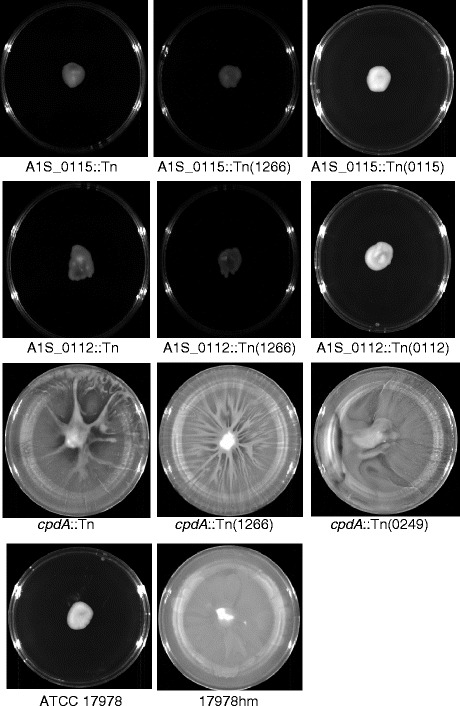
Motility of *A. baumannii* ATCC 17978, 17978hm, and transposon insertion mutants *cpdA*::Tn, A1S_0112::Tn and A1S_0115::Tn. Overnight grown bacterial cells were used to inoculate Luria-Bertani media containing 0.25 % agar; plates were grown overnight at 37 °C. The A1S_0115::Tn, A1S_0115(0115) and A1S_0115(1266) transposon mutant strains and the A1S_0112::Tn, A1S_0112::Tn(0112) and A1S_0112::Tn(1266) transposon mutant strains only show growth at the initial inoculum site at the centre of the plate giving a non-motile phenotype. However, the *cpdA*::Tn, *cpdA*::Tn(0249) and *cpdA*::Tn(1266) transposon mutant strains retained motility. The complemented strain *cpdA*::Tn(0249) displayed a motility pattern almost identical to the 17978hm parent, whereas *cpdA*::Tn and *cpdA*::Tn(1266) displayed a “spoke-like” motility pattern. ATCC 17978 is included as a control displaying a non-motile phenotype

Traditionally, *A. baumannii* has been considered non-motile due to its lack of flagella; however, two forms of motility known as swarming and twitching have now been described in this species [34, 50, 51]. While sliding motility in non-flagellated bacteria can occur by surfactant production in other bacteria [48], twitching motility within *A. baumannii* is mediated by the extension and retraction of type IV pili as a form of surface translocation [5]. We have shown previously that *A. baumannii* strain 17978hm is motile via swarming on LB medium containing 0.25 % agar [34]. Therefore, we applied these conditions to examine the motility phenotypes of the three pellicle-deficient transposon mutant strains. The A1S_0112::Tn and A1S_0115::Tn mutant strains proved to be non-motile as they only grew at the initial inoculum site on the agar plate (Fig. 6). The A1S_0112::Tn and A1S_0115::Tn mutant strains harboring either a wild-type copy of A1S_0112 or A1S_0115 or an empty vector pWH1266 also gave a negative result (Fig. 6). Our data corroborate the findings by Clemmer *et al.* (2011) [47], that the A1S_0112-0118 operon is required for motility as insertions within this operon were found to be non-motile (Fig. 6). However, the loss of motility seen in the transposon mutant strains is not universal because the *cpdA*::Tn variant remained motile despite presenting a visually different motility pattern compared to the 17978hm parent strain (Fig. 6). The *cpdA*::Tn and *cpdA*::Tn(1266) strains presented a spoke like motility pattern, whereas the parent strain formed an even surface film of motile cells. The *cpdA*::Tn(0249) mutant strain returned the motility phenotype to that of the parent covering the 0.25 % agar plate with motile bacterial cells (Fig. 6). As such, motility was not substantially affected, however the manner by which the bacterial cells exercised their motility phenotype in the *cpdA*::Tn strain was altered*.* These results connect the clinical *A. baumannii* strains with the mutant derivatives of the 17978hm strain, in that, all pellicle forming strains including those that form a weak surface film are motile and when the pellicle is disrupted there is either an alteration in the motile phenotype as seen in the *cpdA*::Tn mutant or complete abolishment of motility.

Bacterial adherence is a critical virulence factor and aids in host and surface colonisation. The adherence of the *A. baumannii* mutant strains to human lung epithelial cells (A549) was assessed; all three transposon insertion strains had adherence capabilities that were not significantly different to that of the 17978hm parent strain (data not shown). This suggests that the mechanisms used by *A. baumannii* for pellicle formation are not essential for adherence to A549 human epithelial cells.

### The levels of cAMP in the *cpdA*::Tn mutant strain are significantly increased compared to the 17978hm parent strain

**Fig. 7:**
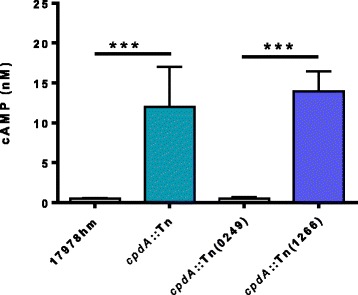
Determination of intracellular cAMP levels. Bacterial cells were grown to an OD_600_ of 0.7, disrupted and cAMP levels determined using the Cyclic Nucleotide XP Enzymatic Immunoassay kit. The amount of cAMP is given in nM representing a sample corresponding to 1 mg of total cellular protein. Bars indicate the standard deviation of three separate experiments. The *cpdA*::Tn transposon mutant has 12 mM of cAMP compared to the parent 17978hm strain which has 0.5 mM of cAMP within the cell. The *cpdA*::Tn(0249) mutant strain returned the cAMP levels to that of the 17978hm parent strain. The *cpdA*::Tn mutant strain harboring the empty vector pWH1266 had no effect on the cAMP levels. Asterisks indicate significant difference (p-value <0.001) as determined by one-way Anova

The maintenance of intracellular cAMP levels is largely a result of controlling *cpdA* expression [52]. Therefore, if *cpdA* is inactivated, cAMP levels are expected to increase in the bacterial cell. Quantification of cAMP levels was achieved using a cyclic nucleotide XP enzymatic immunoassay kit. The *cpdA*::Tn mutant strain was found to have a level of cAMP approximately 24-fold greater than in the 17978hm parent strain (0.5 nM and 12 nM, respectively) (p-value <0.001) (Fig. 7). This confirmed that *cpdA* is involved in cAMP homeostasis within the cell and that the disruption of this gene dramatically affects the accumulation of this secondary signalling molecule. Examination of cAMP levels in strains *cpdA*::Tn(0249) and *cpdA*::Tn(1266) showed that the phenotype could be specifically attributed to the inactivation of *cpdA* (Fig. 7).

### Transcriptional profiling of the *cpdA*::Tn mutant strain under motility conditions

To assess the association between pellicle formation and motility qRT-PCR was undertaken on the *A. baumannii* 17978hm parent and the *cpdA*::Tn transposon mutant strain grown under previously identified motility conditions, i.e. on semi-solid LB agar [43]. Analysis of the operon A1S_0112-0118 (Fig. 3) was undertaken by quantifying the transcription of the A1S_0112 gene, which is the first gene in the operon. In the *cpdA*::Tn strain, the transcription level of A1S_0112 was 2.8-fold down-regulated compared to the 17978hm parent (Table 3). This indicated that CpdA via cAMP plays a role, either directly or indirectly, in regulation of the A1S_0112-0118 operon. This potential regulatory role of CpdA and cAMP on the A1S_0112-0118 operon could explain the altered motility phenotype seen in the *cpdA*::Tn mutant strain, possibly due to alteration in the production of a biosurfactant or through another unknown mechanism(s).

The A1S_0112-0118 operon described above has previously been shown to be activated by quorum-sensing signals in the form of homoserine lactone (*abaI*, A1S_0109) [47]. Furthermore, Saroj and Rather (2013) have shown that down-regulation of *abaI* leads to a reduction in motility in *A. nosocomialis* strain M2 [53, 54]. Therefore, to examine whether the insertional inactivation of *cpdA* in the *cpdA*::Tn mutant had a regulatory effect on the quorum sensing gene *abaI* when grown on motility media, we assessed the levels of transcription of *abaI* using qRT-PCR. The results revealed that, despite the altered motility phenotype of the derivative, *abaI* is expressed at a level comparable to the parent strain (~1.2-fold increase). This suggests that the A1S_0112-0118 operon is regulated independently by two different factors; quorum-sensing (AbaI) and cAMP. Levels of cAMP act as one of the major signalling molecules within the cell and have been show to regulate numerous genes in other bacterial species such as *P. aeruginosa* and *Escherichia coli*, possibly through activation of Vfr [33]. In *P. aeruginosa*, *vfr* is auto-regulated and upon inactivation of *cpdA*, *vfr* expression increases by 12-fold [33, 45]. We investigated this possibility in our *cpdA*::Tn mutant strain by examining the transcript of the putative *vfr* gene (A1S_1182). The qRT-PCR results showed a 1.7-fold decrease in *vfr* expression in the *cpdA*::Tn mutant strain compared to the 17978hm parental strain, that, by a student *t*-test, was determined to be statistically insignificant. Thus, although a 1.7-fold reduction in *vfr* transcription was observed it is not known whether cAMP and Vfr work in concert in *A. baumannii.*

### Whole transcriptome analysis of the CpdA mutant compared to the 17978hm *A. baumannii* parent strain

**Fig. 8:**
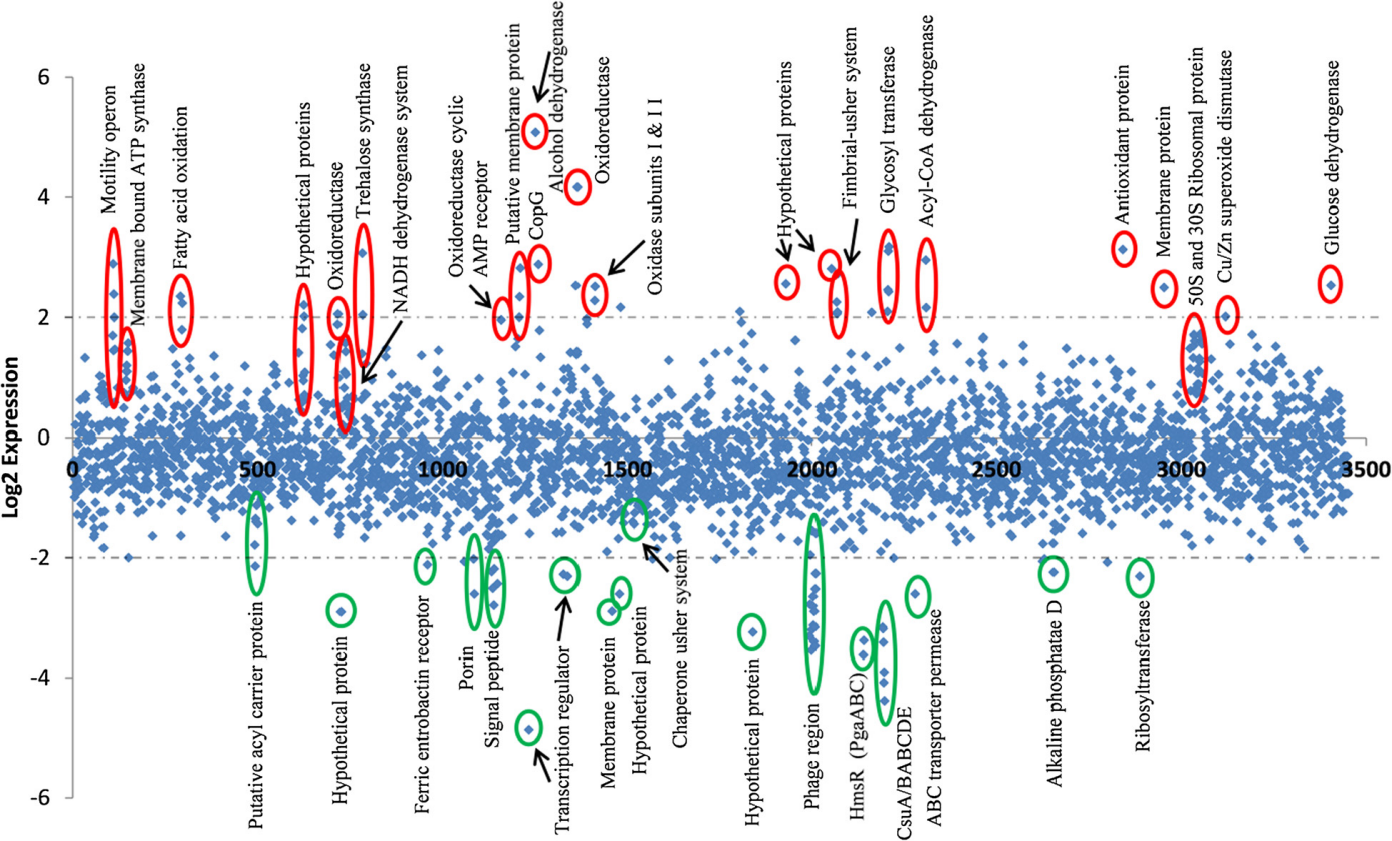
Differential expression of HiSeq RNA data of *cpdA*::Tn and 17978hm strains. Comparative whole transcriptome analysis was undertaken on the *cpdA*::Tn and 17978hm strains using the Illumina platform. Sequence reads were mapped to the reference ATCC 17978 genome and the differential gene expression calculated. Diamond markers indicate the differential expression levels of all predicted open reading frames of the ATCC 17978 genome and are sorted on the x axis according to the locus tag. Changes of 2-fold or more in gene expression were considered significant, up-regulated genes are identified in red and down-regulated genes in green. Dashed lines indicate 4-fold (log_2_ = 2) differences in expression. Genes of particular interest are circled, many of which are discussed in the body of the text

Alterations in the level of intracellular cAMP are known to affect a plethora of phenotypes, including alterations in metabolic processes, biofilm formation and cell surface hydrophobicity [52]. Therefore, RNA sequencing was used to investigate the transcriptome of the *cpdA*::Tn mutant grown in Mueller Hinton broth, in order to assess the impact high levels of cAMP have within the bacterial cell. Transcriptome sequencing revealed that many genes were differentially expressed in the *cpdA*::Tn mutant compared to the parent. Of the approximately 3400 annotated genes in ATCC 17978, 184 genes were more than 2-fold up-regulated and 494 genes were more than 2-fold down-regulated (Fig. 8). The most highly up-regulated gene is identified as an alcohol dehydrogenase (A1S_1274) which showed a 34-fold increase in expression, whereas the greatest down-regulated gene is a putative transcriptional regulator (A1S_1256) showing a 29-fold decrease of expression (Fig. 8).

A number of the genes down-regulated in the *cpdA*::Tn mutant include genes encoding phage-related proteins (A1S_2021-2033), putative acyl carrier proteins, ferric enterobactin receptors, porins, signal peptides, membrane proteins and numerous hypothetical ORFs with no known function (Fig. 8). Variations in the expression of multiple potential transcriptional regulators were also observed; this may be due to the significant alterations that cAMP levels have on the general metabolism of the cell. Genes of particular interest in this study that are down-regulated include the CsuA/BABCDE system (A1S_2213-2218) (9 to 20-fold), the *hms* operon which consists of three genes designated A1S_2160 to A1S_2162) (10 to12-fold), and a chaperone usher system (A1S_1507-1510) (2-fold). Proteins encoded by the *csu* cluster and chaperone usher system have been identified in pellicle and biofilm formations [2, 20, 22, 23, 34, 55, 56]. Interestingly, a single polynucleotide polymorphism leading to truncation of the *csuB* gene potentially inactivating this operon has been identified within ATCC 17978 [2, 22, 56]. Within the *hms* operon two proteins (A1S_2160 and A1S_2161) share an average of 44 % identity with the PgaBC proteins (A1S_0938-0939), the third protein within Hms (A1S_2162) has been designated as a biofilm synthesis protein and shares no homology to the Pga proteins in *A. baumannii* ATCC 17978. The Pga system has previously been identified as being important in biofilm formation [57]. Interestingly, a recent paper by Nait Chabane *et al*. (2014) [23] found that CsuA/BABCDE, PgaABC proteins and the chaperone usher system described above, are over represented within *A. baumannii* strains displaying a pellicle phenotype. This suggests that the Hms and chaperone usher pilus proteins could be key components in the formation of the pellicle matrix.

Numerous genes which are up-regulated encode proteins involved in oxidative phosphorylation (Fig. 8). In particular, these include those encoding ubiquinol oxidase subunit I and II (A1S_1434 and A1S_1433) which are 5.7- fold and 4.9-fold up-regulated, respectively. Other genes involved in oxidative phosphorylation include the NADH dehydrogenase complex (A1S_0762-0764) and the ATP synthetase complex (A1S_0148-0156). One of the most highly up-regulated genes (A1S_1387) is an oxidoreductase (18-fold); this class of enzymes is often involved in the FAD to FADH_2_ conversion, where FADH_2_ plays a major role in oxidative phosphorylation. Therefore, many of the up-regulated genes are involved in the energy, and by extension, the metabolic status of the cell which is not surprising given the central role cAMP plays in cell metabolism.

Other genes of interest which were significantly up-regulated are those involved in 50S and 30S ribosome assembly. The potential role of these up-regulated ribosomal proteins maybe in the assembly of 100S ribosomes, as these are required for cells to survive long term nutrient stress in situations where glucose is low and cAMP is high [58]. Other up-regulated genes including those involved in fatty acid oxidation, trehalose synthase, membrane proteins, dehydrogenase proteins and a number of proteins which are annotated as hypothetical ORFs.

A small operon consisting of 4 genes (A1S_2088-2091) was identified as having 4-fold increase in expression. Proteins encoded by this region have recently been found in extracted pellicle material [23]. Since, *cpdA::*Tn is not capable of pellicle production it would seem unlikely that the A1S_2088-2091 operon is involved in this phenotype, however, *cpdA::*Tn does show an increase in biofilm formation compared to the parent strain and as such this operon may contribute to liquid/surface biofilm production.

The previously described motility operon A1S_0112-0118 [47] is up-regulated approximately 3.8-fold in the *cpdA*::Tn mutant transcriptome, which is in contrast to RT-PCR data (Table 3). These RT-PCR studies were conducted on motility agar to look at the influence of motility on the change in transcription of this operon. On motility agar both cAMP and quorum sensing may play a role in the control of this operon, however in liquid culture grown to mid-log phase used for the RNA-sequence analysis, cAMP appears to be solely responsible for the regulation of A1S_0112-0118.

## Conclusions

This report has shown that pellicle formation is a phenotypic trait seen in a limited number of *A. baumannii* strains. For the first time, the secondary signalling molecule cAMP has been shown to play a critical role in pellicle formation as well as motility in *A. baumannii.* Additionally, this study has shown that insertions in CpdA, A1S_0112 and A1S_0115 completely abolish pellicle formation. Furthermore, transcriptional analyses identified that cAMP effects the expression of the A1S_0112-0118 operon essential for motility, thereby altering the migration pattern seen in the *cpdA*::Tn mutant strain grown on semi-sold agar; insertions in the above operon in A1S_0112 and A1S_0115 resulted in a non-motile phenotype. We thus speculate, that pellicle and motility phenotypes of *A. baumannii* are linked via the expression of cAMP and the A1S_0112-0118 operon. Increased cell surface hydrophobicity was identified as an indicator of pellicle formation ability in the clinical strains tested herein. Whole transciptome analysis has identified an abundance of ORFs affected by the increase in cAMP within the *cpdA*::Tn mutant. A number of these correlate with genes previously known to be involved in pellicle formation including the *hms* locus and a chaperone usher system as well as the A1S_0112-0118 region identified in this study. Considering the importance of pellicle formation in the persistence and transmission of numerous bacterial species, this study has contributed to our understanding of the regulatory mechanisms behind these significant virulence traits in *A. baumannii*.

## Methods

### Bacterial strains

The 54 clinical *A. baumannii* bacterial strains used in this study and the hyper-motile derivative of *A. baumannii* ATCC 17978 designated 17978hm have been described previously [34, 59]. All *A. baumannii* strains were cultured on LB media containing 1 % agar overnight at 37 °C, and single colonies transferred to LB broth for subsequent use in assays, unless otherwise stated. For maintenance, conjugation, cloning and replicating plasmids *E. coli* DH5α, SM10 or JM109 cells were used [60–62].

### Assessment of pellicle formation

The pellicle formation assay was adapted from previously described protocol [35]. In brief, overnight bacterial cultures were diluted 1:100 in 5 mL of LB broth and grown stagnant in polypropylene or glass tubes for 72 h at 25 °C without shaking in the dark [63, 64]. Quantitative measurement of the pellicle material was assessed by the addition of 1 mL of ethanol into the tube underneath the pellicle material. The floating pellicle was then removed and resuspended in phosphate buffered saline (PBS) and the OD_600_ measured using a spectrophotometer (Beckman DU 640). Quantitative data were collected from at least three experiments on three different days.

### Motility assays

Motility was assessed by migration on semi-solid surfaces using media containing 0.25 % agar as described previously [34]. Briefly, an overnight colony was collected using a sterile loop and used to inoculate the centre of LB medium containing 0.25 % agar. The plate was then incubated overnight at 37 °C. Strains were considered motile if any movement of growth from the original inoculation site was observed.

### Eukaryotic cell adherence assays

*A. baumannii* cell adherence to A549 (human type 2 pneumocytes) was investigated as previously described [34]. In brief, cell lines were grown in Dulbecco’s Modified Eagle medium (Invitrogen, Australia) supplemented with 10 % foetal bovine serum (Bovogen, Australia). Washed A549 monolayers in 24-well tissue culture plates were infected with bacterial inoculums containing ~1x10^7^ colony forming units. After incubation at 37 °C for 4 h the culture medium was removed and the monolayers washed with PBS. The cell monolayers were detached from the plate using a treatment of 100 μL of 0.25 % trypsin in PBS. Eukaryotic cells were subsequently lysed by the addition of 400 μL 0.025 % Triton X-100 and serial 10-fold dilutions were spread plated on LB agar to determine the number of colony forming units of adherent bacteria per well. The collated data for the adherence assay were obtained from at least three independent experiments and represent the data points from each experiment in quadruplicate wells.

### Cell surface hydrophobicity tests

Cell surface hydrophobicity was examined as described previously [65]. In brief, overnight cultures were diluted in LB broth and incubated at 37 °C for 2 h. Potassium urea magnesium buffer was used to wash the cells twice, before incubation at 30 °C for 20 min. The cell suspension was adjusted to OD_600_ ~ 0.25 in the same buffer in glass vials before the addition of xylene (3 mL). The sample was vortexed for 20 sec and the aqueous phase transferred to a 1.5 mL tube. The OD_600_ of the cell suspension was determined before (OD_initial_) and after (OD_final_) the addition of xylene. The hydrophobicity index, expressed as a percentage, is calculated as (OD_inital_ − OD_final_/OD_initial_)*100. Quantitative data were collected from at least three experiments from three different days.

### Transposon mutagenesis

Transposon mutagenesis was undertaken using a modified mini Tn*10* transposon (mini::Tn*10*:*gfp*:*kan*) carried on the plasmid pLof [66]. The pLof mini::Tn*10*:*gfp*:*kan* plasmid was transformed into the donor *E. coli* SM10 strain and mated with the *A. baumannii* 17978hm recipient at a 1:10 ratio for 4 h at 37 °C on LB agar exconjugates were resuspended in 5 mL of LB broth and plated onto selective media containing 50 μg/mL kanamycin and 25 μg/mL chloramphenicol to select for transposition events and counter-select against the *E. coli* SM10 donor strain. Approximately, 4000 exconjugates were individually picked using sterile toothpicks to inoculate 200 μL of LB broth in 96 well microtitre plates. The cells were grown at 25 °C in the dark for 72 h and assessed visually for pellicle loss.

### Identification of mini::Tn*10*:*gfp*:*kan* insertion in the chromosome

Chromosomal DNA isolated from *A. baumannii* 17978hm containing the mini::Tn*10*:*gfp*:*kan* was digested with restriction endonucleases *Xba*I and *Sac*II for 4 h at 37 °C. Digested products were cloned into pBluescript SK II (Agilent technologies, CA, USA) and clones were confirmed by sequencing (Australian Genome Research Facility, Adelaide, South Australia). The insertion site of the transposons in the chromosome was determined by Sanger sequencing using an oligonucleotide designed to read out of the end of the transposon (see Additional file 2).

### Complementation of transposon insertions

For genetic complementation of the non-pellicle forming *A. baumannii* mutants, the genes corresponding to A1S_0112, A1S_0115 and *cpdA* were PCR amplified, cloned into the shuttle vector pWH1266, transformed into DH5α *E. coli* cells and sequenced. Plasmids were extracted and subsequently transformed via electroporation using a MicroPulser (Bio-Rad, CA, USA) at 2.5 kV, 200 ohms and 25 microfarads into their respective *A. baumannii* transposon insertion strains [46]. PCR was achieved with VELOCITY™ DNA polymerase (Bioline, Vic, Australia) and the oligonucleotides (Additional file 2) using the following PCR protocols. For amplification of *cpdA*, cycling conditions were 2 min at 94 °C, 30 cycles of 45 sec at 94 °C, 45 sec at 55 °C, and 90 sec at 72 °C, followed by a final extension of 4 min at 72 °C. Amplification of A1S_0112 used the same conditions with the exception of the annealing temperature which was increased to 60 °C. For amplification of A1S_0115, the cycling conditions were 2 min at 94 °C, 30 cycles of 90 sec at 94 °C, 90 sec at 60 °C, 4 min at 72 °C and a final extension of 6 min at 72 °C.

### Measurement of intracellular cAMP levels

Assessment of cAMP levels within *A. baumannii* strains *cpdA*::Tn, *cpdA*::Tn(0249), *cpdA*::Tn(1266) and 17978hm was performed with cells were grown to an OD_600_ of 0.7. Cells were pelleted by centrifugation at 4500 *g* for 15 min, resuspended in PBS and disrupted at 30,000 lb per square inch using the One Shot Head from the TS series bench top disruptor (Constant Systems, United Kingdom). The resulting cellular material in the cell lysis solution was clarified by centrifugation at 4500 *g* for 30 min and the supernatant removed and stored at −80 °C until required. The concentration of cAMP was measured using the Cyclic Nucleotide XP Enzymatic Immunoassay kit (Cell Signalling Technology, MA, USA) according to the manufacturer’s instructions. In brief, 50 μL of horseradish peroxidase-linked cAMP solution and 50 μL of sample were added to the supplied 96-well tray, which was then covered and incubated at room temperature for 3 h on a horizontal orbital plate shaker. The contents of the plate were discarded and the plate was washed 4 times with 200 μL/well of wash buffer. Next, 100 μL of 3,3′,5,5′-tetramethylbenzidinesubstrate solution was added and incubated for 30 min at room temperature, after which 100 μL of stop solution was added and the absorbance measured at 450 nm. Using the inverse for the standards derived from a standard curve and adjusted for the amount of total protein added, the amount of cAMP was calculated. Total protein concentrations were quantified using the Lowry protein assay (Bio-Rad, CA, USA) following the manufacturer’s instructions.

### Isolation of total cellular RNA

To measure transcriptional levels for either RT-PCR or in Hiseq RNA transcriptome analysis conducted by SA Pathology (Central Adelaide local Health Network via an Illumina platform), cells were isolated and RNA extracted as below. Bacterial cells were harvested from either semi-solid media (0.25 % LB agar) for RT-PCR using pre-chilled PBS or harvested from Mueller Hinton broth at OD_600_ of 0.6 for Hiseq. Bacterial cells were pelleted by centrifugation at 4500 *g* for 15 min and lysed in 1 mL TRIzol reagent (Life Technologies, Australia) and 200 μL of chloroform. Following phase separation, the aqueous layer was collected and RNA isolated using the Isolate RNA Mini Kit (Bioline, NSW, Australia) following the manufacturer’s recommendations. Samples for RT-PCR were subsequently treated with DNaseI (Promega, WI, USA) for 30 min at 37 °C and 1 μL of stop solution was added before a final incubation for 10 min at 65 °C. Triplicate samples were pooled and sent to SA Pathology for Ribosomal RNA reduction using Epicentre Ribo-Zero kit and RNA library bar-coding was undertaken on RNA for HiSeq. Samples were run on the Illumina HiSeq platform (1X50 base pair single reads). These data are accessible through GEO Series accession number GSE64935 (http://www.ncbi.nlm.nih.gov/geo/query/acc.cgi?acc= GSE64935).

### Bioinformatic analysis

The quality of the cDNA reads obtained from the HiSeq was checked using ‘Fastqc’ (http://www.bioinformatics.babraham.ac.uk/projects/fastqc/). Reads were subsequently mapped to the reference genome using ‘bowtie’ aligner (http://bowtie-bio.sourceforge.net/index.shtml). For the reads obtained, approximately 95 % were mapped to the ATCC 17978 genome of which 78 % mapped to the coding regions. In order to determine changes in gene expression the number of reads obtained for each ORF was normalised using reads per kilobase of transcript per million reads mapped.

### Quantitative reverse transcription PCR

M-MLV reverse-transcriptase (Promega) and random hexamers (GeneWorks, SA, Australia) were used to synthesise complementary deoxyribonucleic acid (cDNA) following the manufacturer’s recommendations. qRT-PCR was performed on three biological replicates using DyNAmo SYBR green qRT-PCR kits (ThermoScientific, Vic, Australia) in conjunction with a Rotor-Gene RG-3000 (Corbett Life Science, Australia). A typical qRT-PCR run was as follows: 1 min at 95 °C, 40 cycles of 10 sec at 95 °C, 15 sec at 55 °C and 20 sec at 72 °C [67]. Transcriptional differences were calculated using the ΔΔC_T_ method [68]. The 16S rRNA (A1S_r01) and *gapDH* (A1S_2501) transcription levels were used as a reference.

## Additional files

Additional file 1:
**Position of the Tn** ***10:kan:gfp*** **insertion within the** ***A. baumannii*** **17978hm non-pellicle forming mutants.**
Additional file 2:
**Oligonucleotides used for cloning, sequencing and qRT-PCR.**
Additional file 3:
**Growth curve of the transposon insertion mutants.** *A. baumannii* insertion mutants were subcultured 1:100 dilution into fresh Luria-Bertani broth; 200 μL samples were taken every hour and the OD_600_ determined spectroscopically.
