# Supplementary material for: Identification of genes essential for pellicle formation in Acinetobacter baumannii
Source: BMC Microbiol. 2015 Jun 6;15:116. doi: 10.1186/s12866-015-0440-6 (PMC4457973; doi:10.1186/s12866-015-0440-6)
Supplement: Additional file 1: — Position of the Tn 10:kan:gfp insertion within the A. baumannii 17978hm non-pellicle forming mutants. [file 12866_2015_440_MOESM1_ESM.pdf]

Additional file 1

Position of the Tn*10:kan:gfp* insertion within the *A. baumannii* 17978hm non-pellicle forming mutants

Mutant	Position within the chromosome[a]	Open reading frame	Description given by KEGG[b]
1	275449	A1S_0249	Cyclic 3'5'-adenosine monophosphate phosphodiesterase
2	137827	A1S_0115	Amino acid adenylation
3	137821	A1S_0115	Amino acid adenylation
4	137827	A1S_0115	Amino acid adenylation
5	134323	A1S_0112	Acyl-CoA synthetise/AMP-acid ligases II
6	137819	A1S_0115	Amino acid adenylation
7	137827	A1S_0115	Amino acid adenylation
8	137827	A1S_0115	Amino acid adenylation
9	139814	A1S_0115	Amino acid adenylation

[a] Positions are relative to *A. baumannii* ATCC 17978 GenBank (CP000521)

[b] KEGG: Kyoto Encyclopedia of genes and genomes; www.genome.jp/kegg
