# Supplementary material for: Identification of genes essential for pellicle formation in Acinetobacter baumannii
Source: BMC Microbiol. 2015 Jun 6;15:116. doi: 10.1186/s12866-015-0440-6 (PMC4457973; doi:10.1186/s12866-015-0440-6)
Supplement: Additional file 2: — Oligonucleotides used for cloning, sequencing and qRT-PCR. [file 12866_2015_440_MOESM2_ESM.pdf]

Additional file 2

Oligonucleotides used for cloning, sequencing and qRT-PCR

Name /Target	Forward 5'–3'	Reverse 5'–3'
**A1S_0112comp**	[a]GAGAgaattcGCAACTTTGGAATCCCGCGCA	[a]GAGAggatccTTGCTCCAGACGAGCAGCAG
**A1S_0115comp**	[a]GAGAgaattcTTCTGAGTTGCAGCAGGGAG	[a]GAGAggatccAACCTGCATCATTGGCTGGG
**A1S_0249**	[a]GAGAggatccCTGTTCGATGAAAATCACCC	[a]GAGAggatccCTTTAGGGCTAATAACCTG
**Tn10- F2**	CGGCCGCACGCGTATTCAGG	
**A1S_r01 (16SrRNA)**	CAGCTCGTGTCGTGAGATGT	CGTAAGGGCCATGATGACTT
**A1S_2501 (GapDH)**	CAACACTGGTAAATGGCGTG	ACAACGTTTTCATTTCGCC
**A1S_0109**	TCTGGTGAGCAGGGATAGG	TGTCGTGGCTCAAGACAGAG
**A1S_0112**	ACGCCAGTCTGGTGGTATTC	AGGTTCGAACAGCAATACGG
**A1S_0115**	GCGGTCAAAATCATTATGTG	GCTCTGCTGCTTATCGGCAT
**A1S_0118**	GGACTCTCTATTATCGGACG	CAGATAAATCGCTCAAGCTGC

[a]Nucleotides indicated by the lowercase letters indicate the restriction sites used for cloning
