# Supplementary material for: Identification of genes essential for pellicle formation in Acinetobacter baumannii
Source: BMC Microbiol. 2015 Jun 6;15:116. doi: 10.1186/s12866-015-0440-6 (PMC4457973; doi:10.1186/s12866-015-0440-6)
Supplement: Additional file 3: — Growth curve of the transposon insertion mutants. A. baumannii insertion mutants were subcultured 1:100 dilution into fresh Luria-Bertani broth; 200 μL samples were taken every hour and the OD600 determined spectroscopically. [file 12866_2015_440_MOESM3_ESM.pdf]

Additional file 3

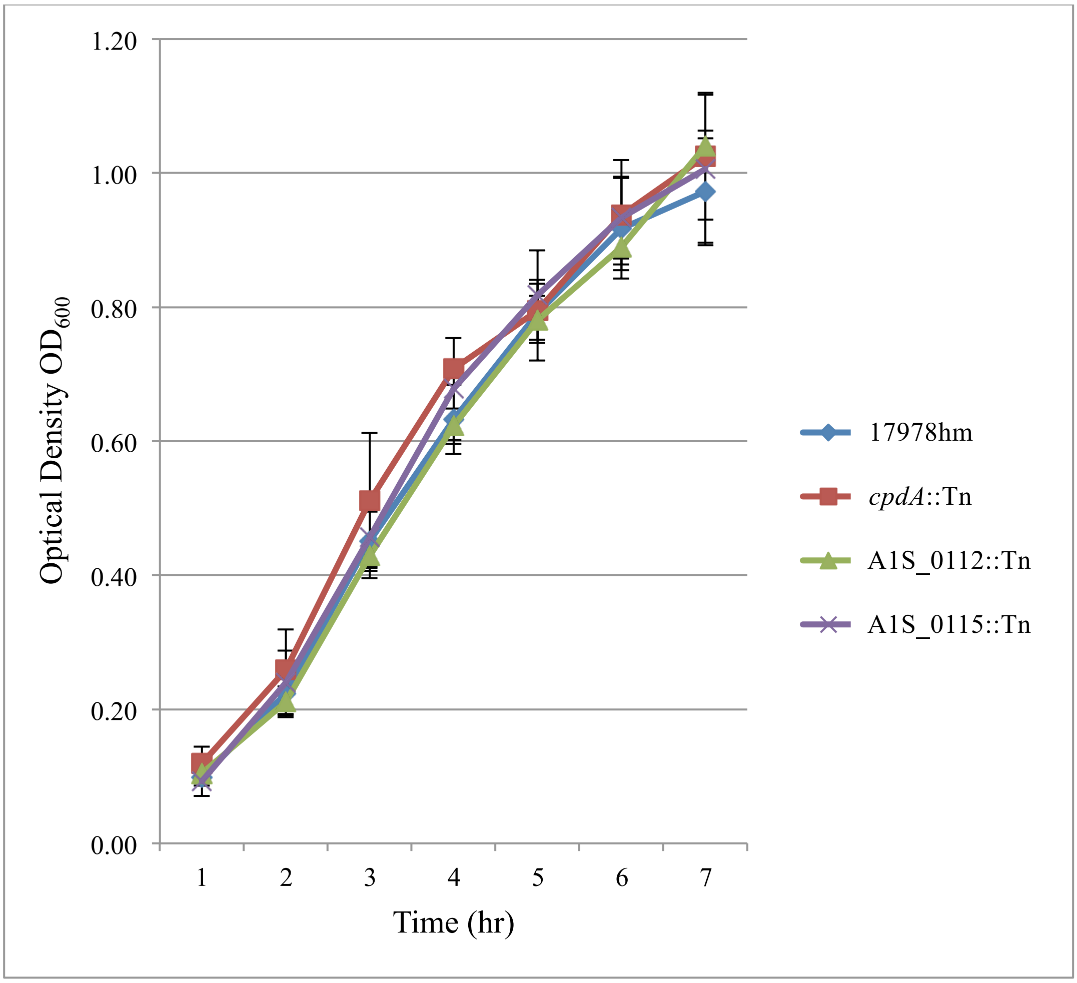

Growth curve of the Tn*10* insertion mutants. *A. baumannii* 17978hm transposon insertion mutants were subcultured 1:100 dilution into fresh Luria-Bertani broth; 200 µl samples were taken every hour and the OD_{600} determined spectroscopically.
